# Goltz syndrome in males: A clinical report of a male patient carrying a novel *PORCN* variant and a review of the literature

**DOI:** 10.1002/ccr3.1783

**Published:** 2018-09-21

**Authors:** Sofia Frisk, Catherine Grandpeix‐Guyodo, Karin Popovic Silwerfeldt, Helgi Thor Hjartarson, Dimitris Chatzianastassiou, Irina Magnusson, Tobias Laurell, Ann Nordgren

**Affiliations:** ^1^ Department of Molecular Medicine and Surgery Center of Molecular Medicine Karolinska Institutet Stockholm Sweden; ^2^ Department of Clinical Genetics Karolinska University Laboratory Karolinska University Hospital Stockholm Sweden; ^3^ Department of Dermatology Danderyd Hospital Stockholm Sweden; ^4^ Department of Neuropediatrics Astrid Lindgren Children's Hospital Stockholm Sweden; ^5^ Department of Clinical Pathology Karolinska University Hospital Stockholm Sweden; ^6^ Department of Hand Surgery Södersjukhuset Stockholm Sweden

**Keywords:** focal dermal hypoplasia, Goltz syndrome, male, *PORCN*

## Abstract

Here, we report a novel mosaic mutation in the *PORCN* gene in a male Goltz syndrome patient. We also compare the phenotypes of all reported males with a confirmed molecular diagnosis. This report serves to further clarify the phenotype of Goltz syndrome and suggests that expression in males varies.

## Graphical abstract

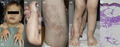

## 1 INTRODUCTION

Goltz syndrome (GS), also known as Focal dermal hypoplasia (OMIM #305600), is a rare X‐linked dominant syndrome with variable meso‐ectodermal abnormalities.^1^ Common clinical findings concern the skin (patchy skin aplasia, subcutaneous fat herniation, papilloma, telangiectasia, sparse hair, syndactyly, dysplastic nails, linear hypo‐/hyperpigmentation often following the lines of Blaschko), skeleton (ectrodactyly, oligodactyly, transverse limb defects, long bone reduction defects), eyes (anophtalmos, microphthalmia, cataract, choroid or retinal coloboma), and face (facial asymmetry, hypoplastic alae nasi, abnormal ear morphology, and dental defects).^2^

The molecular basis of GS was first described in 2007, when it was reported that GS is caused by loss‐of‐function mutations in the *PORCN* gene.^3^, ^4^ *PORCN* is the human homolog of a Drosophila polarity gene “porcupine”, located on the X chromosome (Xp11.23). The *PORCN* gene encodes a 461‐amino acid 52‐kDa endoplasmic reticulum protein, the porcupine protein, thought to be important for modification and excretion of Wnt proteins.^5^, ^6^ Wnt proteins are critical for interactions between ectoderm and mesoderm during embryogenesis.^4^, ^7^ From what is known today, *PORCN* is the only gene associated with GS. A total of 171 different mutations are registered in the online *PORCN* mutation database (http://www.lovd.nl/PORCN. Accessed on February 6, 2018), and these are scattered throughout the entire coding sequence of the *PORCN* gene.^8^

The phenotype in GS male patients is not extensively depictured, and it has not been reported how many male patients exist in total with a confirmed molecular diagnosis. This report aims to further clarify the genotype‐phenotype correlation of GS in males, which is important since male patients might have been missed or misinterpreted.

## 2 MATERIALS AND METHODS

### 2.1 Study approval

The study was performed in accordance with the Declaration of Helsinki, and the local ethical board in Stockholm approved the study. Informed consent was obtained from the family according to local ethical guidelines, and the publication of clinical data and pictures has been approved by the parents.

### 2.2 Genetic analysis

Genomic DNA was extracted from blood lymphocytes and fibroblasts from skin biopsies from affected and normal skin by standard protocols. The *PORCN* gene was amplified by PCR and screened for mutations by genomic sequencing of both DNA strands of the entire coding region and the highly conserved exon‐intron splice junctions. We performed digital PCR (QuantStudio 3D; Applied Biosystems, Foster City, CA, USA) using the manufacturer's protocol. For array comparative genomic hybridization (CGH), a custom 4x180K array CGH platform was used (Oxford Gene Technologies, Oxfordshire, UK). This platform has a genomewide average base pair spacing of about 20 Kb. Experiments were performed according to the manufacturer's protocol.

## 3 RESULTS

### 3.1 Clinical report

**Figure 1:**
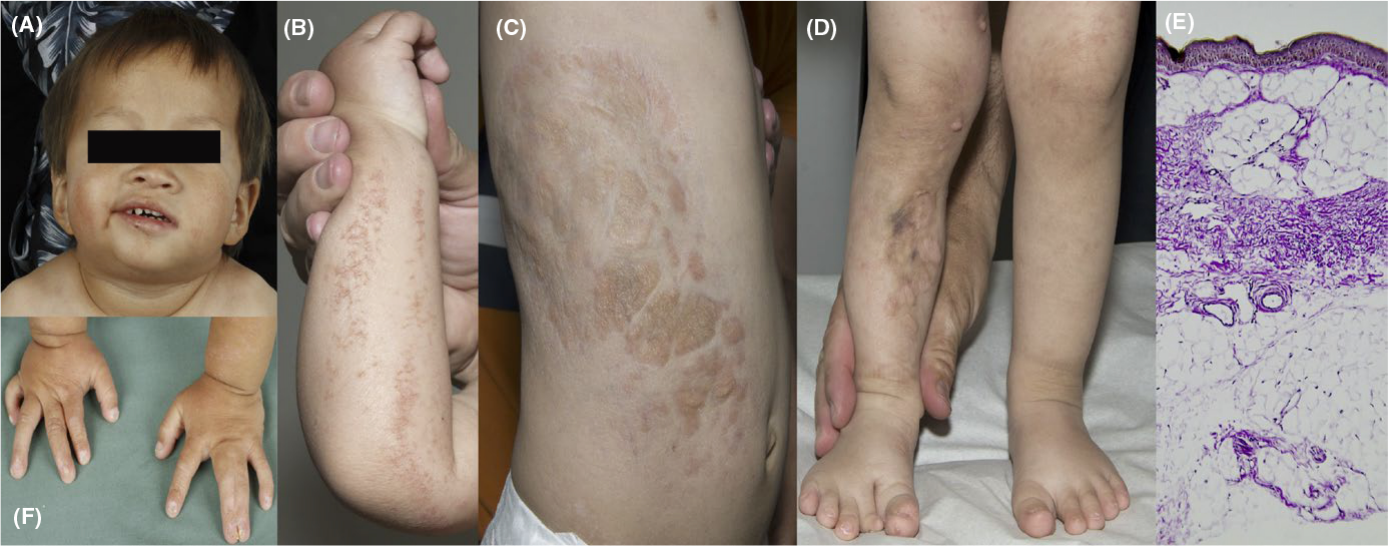
A, Right‐sided facial hypoplasia. Widely spaced teeth and low set ears. B, Hypopigmentation in a Blaschkolinear distribution flanked by telangiectasia on the right arm. C, Atrophic skin with nodular fat herniation clustered on the right‐side trunk. D, Patchy skin aplasia on the right lower leg. Partial ectrodactyly of the second toe. E, Slightly atrophic epidermis lacking adnexal structures. Collagen bundles greatly diminished and replaced by mature adipose tissue in dermis, reaching up to the epidermis in a “nevus lipomatosus superficialis‐like” manner. F, Clinodactyly on both hands and a complete syndactyly of the third and fourth fingers of the left hand. Ridged nails of all fingers of the left hand and of the second and the third finger of the right hand.

A 3‐year‐old male was referred to the Department of Dermatology at Danderyd Hospital in Stockholm to explore multiple skin aberrations. The patient had been adopted from China, and his medical history was unknown. The patient had several abnormalities: His face was asymmetric with a right‐sided hypoplasia, widely spaced teeth, and low set ears (Figure 1A). There was hypopigmentation in a blaschkolinear distribution on his right arm (Figure 1B). Telangiectasia was present on both arms (Figure 1B) and on the left leg (Figure 1D) in a blaschkolinear distribution. He had atrophic skin with nodular fat herniation clustered on the right‐side trunk (Figure 1C). There were areas of patchy skin aplasia on his right leg (Figure 1D). His right foot showed partial ectrodactyly of the second toe (Figure 1D). He had clinodactyly on both hands and a complete syndactyly of the third and fourth fingers of the left hand (Figure 1E). All the nails of the left hand and the nails of the second and third finger of the right hand were ridged. In addition, he had a right‐sided dacryostenosis, short stature (−4 SD), inguinal hernia, and osteopathia striata. A diagnosis of GS was suspected clinically.

### 3.2 Histopathological findings

Skin biopsies were performed on the atrophic patches, and microscopy showed thinning of dermis, few collagen structures, and herniation of fat lobules in the upper dermis (Figure 1E).

### 3.3 Genetic findings

A previously unreported mosaic variant in exon 14, c.1274_1275del (p.Thr425Argfs*45) (NM_203475.2), was identified in samples of blood, affected skin, and normal skin by Sanger sequencing. To quantify the mosaic levels in each tissue, we performed digital PCR which showed the variant in 72% of the cells in affected skin, 49% in normal skin, and 47% in blood. The variant creates a frame shift starting at codon Thr425. The new reading frame ends in a stop codon 44 positions downstream. This *PORCN* variant was classified as likely pathogenic. Array CGH was normal and excluded Klinefelter syndrome**.**

## 4 DISCUSSION

This paper describes a mosaic GS male patient with a previously unreported *PORCN* variant and gives an overview of genetic and clinical findings in all male patients. To the best of our knowledge, this is the 24th GS male patient reported with a confirmed molecular diagnosis.

The clinical presentation of our patient is typical for GS and in accordance with the proposed diagnosis criteria.^2^ In addition, the detected variant leads to a stop codon, which makes the diagnosis GS very likely. However, mosaic levels were also found in unaffected tissue. We conclude that the mutation exists in both affected and unaffected tissue, but in a higher percentage in affected tissue. It has been observed in other mosaic disorders that the mutation percentage may vary and that mutations can occur even in seemingly unaffected tissue.^9^, ^10^

When reviewing the literature, we found more than 400 reported GS patients.^11^, ^12^ GS was long presumed to be lethal in males.^13^, ^14^ However, later reports have shown that approximately 10% of all GS patients are males.^2^, ^15^ The majority of surviving males can be explained by mosaicism for *PORCN* mutations that arise postzygotically during embryogenesis 16 or Klinefelter syndrome, since these males carry an additional X chromosome.^5^ There is no genotype‐phenotype correlation, which can be explained by random X‐inactivation in females or mosaicism.^17^, ^18^, ^19^ Of the 24 reported male patients, 19 are mosaics, one patient has Klinefelter syndrome, and four patients from two different families are non‐mosaics (Table 1). Interestingly, all four patients with non‐mosaic *PORCN* mutations lacked skin manifestations but had other severe symptoms compatible with GS.^20^, ^21^ In addition, diaphragm abnormalities were detected in two patients, resembling previously reported cases with pentalogy of Cantrell.^18^, ^22^

**Table 1:** Overview of genetic and clinical features in all reported GS male patients with a confirmed *PORCN* mutation

Patient (reference)	1 (Madan et al)^21^	2 (Madan et al)^21^	3 (Brady et al)^20^	4 (Brady et al)^20^	5 (Alkindi et al)^5^	6 (Durack et al)^7^	7 (Rao et al)^19^	8 (Yoshihashi et al)^16^	9 (Stevenson et al) 25	10 (Vreeburg et al)^13^	11 (Bornholdt et al)^15^	12 (Bornholdt et al)^15^	13 (Bornholdt et al)^15^	14 (Bornholdt et al)^15^	15 (Maas et al)^18^	16 (Wang et al)^4^, ^12^	17 (Wang et al)^4^, ^12^	18 (Wang et al)^4^, ^12^	19 (Wang et al)^4^, ^12^	20 (Bostwick et al)^2^	21 (Bostwick et al)^2^	22 (Peters et al)^1^	23 (Young et al)^14^	24Our patient	Frequency
Genotype	c.749C>T		c.470G>A		c.1093C>T 46,XXY	c.1039_1046delinsT	c.898G>T	c.129G>A	c.956dup	c.886del	c.502G>A	c.1110del	c.1186C>T	c.1315T>C	c.571C>T	c.1059_1071dup	c.370C>T	c.1064_1081del	c.1093C>G	c.956dup	c.1059_1071dup	c.853_855del	c.956dup	c.1274_1275del	–
Exon	9		5		13	12	10	2	11	10	5	13	14	15	6	12	4	12	13	11	12	10	11	14	–
Protein alteration	p.Ser250Phe		p.Gly157Asp		p.Arg365Trp	p.Leu347Trpfs*50	p.Glu300*	p.Trp43*	p.Asn320Glufs*99	p.Arg296Glyfs*18	p.Gly168Arg	p.Ile371Serfs*28	p.Arg396*	p.Trp439Arg	p.Gln191*	pThr358Profs*65	p.Arg124*	p.Ala355_Val360del	p.Arg365Gly	p.Asn320Glufs*99	pThr358Profs*65	p.Thr285del	p.Asn320Glufs*99	p.Thr425Argfs*45	–
Mosaic	−	N/A	N/A	−	−	+	+	+	+	+	+	+	+	+	+	+	+	+	+	+	+	+	+	+	19/24
Birth weight (g)	1900	N/A	3010	3040	N/A	N/A	2400	3290	N/A	1050	N/A	N/A	N/A	N/A	N/A	N/A	N/A	N/A	N/A	N/A	N/A	2700	N/A	N/A	−
Typical skin/hair findings	−	−	−	−	+^1^	+^2^	+^3^	+^4^	+^5^	+^6^	+^7^	+^7^	+^8^	+^7^	+^9^	+^10^	+^11^	+^12^	+^11^	+^13^	+^14^	+^15^	+^16^	+	20/24
Microphthalmia	+	+	+	+	+	−	−	−	−	−	−	−	−	−	−	−	−	−	+	+	−	−	−	−	7/24
Coloboma	+	−	+	−	+	−	+	−	+	−	+	−	−	−	−	−	−	−	+	+	−	+	+	−	10/24
Other ocular defects	+^17^	−	−	+^18^	+^19^	−	−	+	+^20^	−	−	−	+^21^	−	−	−	−	−	−	−	−	+^22^	+	+^23^	9/24
Any ocular defect	+	+	+	+	+	−	+	+	+	−	+	−	+	−	−	−	−	−	+	+	−	+	+	+	15/24
Dental defects	N/A	N/A	−	−	+^24^	+^25^	+^26^	−	−	−	+	−	+	−	−	+	−	−	−	+	+	−	−	+	9/24
Syndactyly	+^27^	+^28^	+	+	+^28^	−	+^29^	+^30^	+^31^	+^32^	+	−	+	+	−	−	+	+	+	+^29^	+^33^	+^34^	+	+	20/24
Ectrodactyly	+^29^	−	−	−	+^33^	−	+	−	+^33^	−	−	−	−	−	−	+	+	−	+	+^35^	+^36^	+^37^	+^38^	+	12/24
Dysplastic nails	+	N/A	−	−	+	−	−	+	+	+	−	−	−	−	−	−	−	+	+	+	+	+^39^	+	+	12/24
Osteopathia striata	+	N/A	−	−	−	−	−	−	+	−	−	−	−	−	−	−	−	+	−	N/A	N/A	−	N/A	+	4/24
Clavicular dysplasia	+	+^40^	−	−	−	−	−	−	−	−	−	−	−	−	−	−	−	−	−	N/A	N/A	−	−	−	2/24
Costovertebral defect	+	−	−	−	−	−	−	−	−	−	−	−	−	−	−	−	−	−	+	N/A	N/A	−	−	−	2/24
Diaphrapmatic hernia	−	+	+	−	−	−	−	−	−	−	−	−	−	−	−	−	−	−	−	N/A	N/A	−	−	−	2/24
Inguinal hernia	−	−	−	−	−	−	−	−	−	+	−	−	+	−	−	−	−	−	−	N/A	N/A	−	−	+	3/24
Cardiac anomalies	+^41^	+	+^42^	+	−	−	−	−	+^43^	−	−	−	−	−	−	−	−	−	−	N/A	N/A	+^44^	+^42^	−	7/24
Pulmonary hypertension	−	−	−	−	−	−	−	−	+	−	−	−	−	−	−	−	−	−	−	−	−	−	+	−	2/24
Brain abnormality	N/A	N/A	N/A	+^45^	+	−	−	−	−	+^46^	−	−	+^47^	−	−	−	−	−	−	N/A	N/A	+^48^	−	−	5/24
Renal anomaly	+^49^	+^50^	−	+^51^	+	−	−	+^51^	−	−	−	−	−	−	−	−	−	−	−	N/A	N/A	−	−	−	5/24
Dysmorphic ears	+^52^	−	−	−	+	−	−	−	+^53^	+^54^	−	−	−	−	−	−	−	−	−	+	−	+^55^	+^56^	+	8/24
Dysmorphic/asymmetric facial features	+^57^	−58	−	−	−	−	+^59^	−	+^60^	−	−	−	−	−	−	−	−	−	−	−	+^61^	−	−	+	5/24

+ =present, ‐ =absent, N/A =not available information.

^1^Red‐yellow atrophic cutaneous streaks with some telangiectasia in a linear pattern following Blaschko's lines on both arms and legs, the posterior neck, and the scalp, with some areas of alopecia. Clustered papillomas on the chin.

^2^Linear, reticulate, atrophic, and erythematous patches on the arms, thighs, and hips along the lines of Blaschko, with fat herniation in the right axilla.

^3^Atrophic areas of skin with yellowish nodules over depigmented macules following the lines of Blaschko. Sparse hair.

^4^On the right occipital scalp, an atrophic macule with small whitish spots and hair loss. Skin depressions <1 cm in diameter on the back and the right buttock. Small whitish depigmented spots, which were slightly depressed from the skin surface, distributed linearly on the trunk and arms. Streaks of brown‐pigmented macules on the dorsal aspect of the legs. Linear brown pigmentations on the dorsal aspect of the legs. Both the linear arrangement of the whitish spots and the streaks of pigmented macules followed the lines of Blaschko.

^5^Cutis aplasia neighboring the anterior fontanelle. Focal dermal hypoplasia following the lines of Blaschko on the lower extremities and linear lesions bilaterally on the trunk. Focal dermal hypoplasia on his right inner thigh. Small papules of the fourth toe and a small mobile mass on the posterior scalp. Sparseness of hair and eyelashes.

^6^Linear erythematous slightly atrophic skin lesions on the left cheek. Linear alopecia on the occipital area, following Blaschko's lines. Further atrophic slightly erythematous macules, sometimes containing telangiectasias, following Blaschko's lines on both flanks and the lateral aspects of both lower legs.

^7^Linear skin lesions.

^8^Linear skin lesions, patchy hairlessness.

^9^Aplasia cutis.

^10^Dermal hypoplasia, blaschkolinear pigmentation.

^11^Dermal hypoplasia.

^12^Dermal hypoplasia, blaschkolinear pigmentation, sparse hair.

^13^Hyperpigmentation, fat herniations, skin atrophy, telangiectasia.

^14^Hyperpigmentation, skin atrophy, telangiectasia.

^15^Hypoplasia, atrophy and linear hypopigmentation following the lines of Blashchko.

^16^Cutis aplasia, dermal hypoplasia.

^17^Microcornea.

^18^Dense intraocular tissue.

^19^Optic nerve atrophy and displaced lenses.

^20^Smaller left eye and subnormal visual evoked potentials in both eyes. Retinal flap near the ora serrata of the right eye.

^21^Optic atrophy.

^22^Bilateral nasolacrimal duct obstruction.

^23^Right side nasolacrimal duct obstruction.

^24^Widely spaced and some missing teeth.

^25^Very few remaining teeth, misshapen, and discoloured.

^26^Oligodontia.

^27^The left hand had syndactyly with a total of three digits. Syndactyly of the right first and second toes and the left third and fourth toes.

^28^Third and fourth toes bilaterally.

^29^Right hand.

^30^Cutaneous syndactyly of the right third and fourth fingers and toes and the left second and third toes.

^31^Syndactyly of the second and third right fingers.

^32^Cutaneous syndactyly of the second and third right fingers.

^33^Right foot.

^34^Both hands.

^35^Right hand and right foot.

^36^Left hand and left foot.

^37^Right hand and foot.

^38^Foot.

^39^Mild.

^40^Pseudo arthrosis of the right clavicle.

^41^Bicuspid aortic valve.

^42^Atrial septal defect.

^43^Small secundum atrial septal defect with spontaneous closure at one year of age, patent ductus arteriosus.

^44^A patent foramen ovale and an aberrant right subclavian artery were identified on echocardiogram.

^45^Enlarged ventricles, partial agenesis of the corpus callosum, and several intracerebral haemorrhages.

^46^Intraventricular right side cyst.

^47^Microcephaly, seizures.

^48^Microcephaly, myelomeningocele, Arnold‐Chiari malformation, and hydrocephalus.

^49^Multicystic kidney dysplasia with renal failure.

^50^Dysplastic kidneys, hydronephrosis, and renal failure.

^51^Hydronephrosis of the left kidney.

^52^Unfolded helices.

^53^Underdevelopment of the superior helices, slightly posteriorly rotated and large relative to his body.

^54^Asymmetric ears, the helix of the left ear showing cranial notching. The left ear slightly cup‐shaped.

^55^Underfolded ears with hypopigmentation of the helices.

^56^Simplified ears with underdevelopment of the superior helices.

^57^Bilateral clefts of the lip and cleft palate.

^58^Narrow face, midface hypoplasia, broad nasal bridge, small mandible.

^59^Right side hemihypotrophy.

^60^Slightly broad nasal tip with unusual linear erythema and dermal hypoplasia at the junction of the alae nasi, and asymmetry of the upper lip with hypoplastic tissue on the left.

^61^Right‐sided facial features more prominent than left‐sided.

When reviewing the literature of GS male patients with a confirmed molecular diagnosis, we noticed that skin findings, syndactyly, ocular defects, and ectrodactyly are common clinical features (Table 1). A study from 2016, including 18 patients with GS, demonstrated a higher incidence of ophthalmologic manifestations in patients with GS than previously reported (77% vs 40%).^23^ In our review over reported male patients, we found that 15 out of 24 (62.5%) had ocular manifestations (Table 1). However, ocular defects are not part of previously suggested clinical diagnosis criteria.^2^ Our suggestion is that GS should be suspected even in the absence of skin findings, if other typical signs occur, such as characteristic limb malformations or ocular defects, including coloboma or microphthalmia. Ocular manifestation should be considered as a major sign of GS, which has been suggested earlier.^24^

In summary, GS is a rare multisystem disorder with highly variable expressivity. This report serves to further clarify the phenotype of GS in males, which is important since there are, to our knowledge, only 24 male patients described with a confirmed molecular diagnosis. The highly variable expressivity might have caused male patients to be missed or misinterpreted until today. Our report adds to the knowledge of genotype‐phenotype correlations in male patients with *PORCN* mutations and highlights that *PORCN* mutations can be suspected in patients with characteristic limb malformations, such as ectrodactyly, or ocular manifestations, even in the absence of characteristic skin findings.

## AUTHORSHIP

SF: coordinated the study, performed the genetic studies and interpreted the data. SF has reviewed the literature and written the draft of the manuscript with input from the other co‐authors. CGG: designed the study, assessed the clinical findings and has been involved in reviewing the literature, drafting the manuscript and revising it critically. KPS: contributed to the study design, assessment of clinical findings and manuscript revision. HTH: contributed to the study design, assessment of clinical findings and has been revising the manuscript critically. DC: performed the histopathological analysis and interpreted the findings. DC has contributed to the figure design and manuscript revision. IM: contributed to the study design, assessment of clinical findings and manuscript revision. TL: contributed to the study design, assessment of genetic findings, figure design and manuscript revision. AN: designed and coordinated the study. AN assessed the genetic findings and has been involved in drafting the manuscript and revising it critically. All authors have read and given final approval for the manuscript.

## CONFLICT OF INTEREST

None declared.
